# DNA microarray analysis reveals variability of accessory genes among isolates recovered during an MRSA outbreak

**DOI:** 10.1186/1753-6561-5-S6-P178

**Published:** 2011-06-29

**Authors:** P Basset, S Monecke, D Blanc

**Affiliations:** 1 Hospital preventive medicine, Centre Hospitalier Universitaire Vaudois, Lausanne, Switzerland; 2 Institute for Medical Microbiology and Hygiene, Dresden University of Technology, Dresden, Germany; 3 Alere Technology, Jena, Germany

## Introduction / objectives

Although MRSA outbreaks might have significant consequences on patients and/or health care resources, few data are available about the microevolution of a strain during a single outbreak. In this study, our aim was to characterize the variability of accessory genes among isolates recovered during a local MRSA outbreak.

## Methods

Ten MRSA isolates recovered during a large outbreak occurring at the Lausanne University Hospital (Switzerland) were characterized using a DNA microarray (StaphyType, Alere Technologies, Germany) that targets approximately 180 genes including several resistance and virulence genes. Each isolate belonged to the South German clone (ST-228-SCC*mec* I) and had been recovered between September 2008 and December 2009.

## Results

As expected during the clonal dissemination of a strain, the 10 isolates shared identical presence/absence for most of the *c.a.* 180 genes tested. Nevertheless, variation was observed for several resistance and virulence genes. These included the beta lactamase operon genes (*blaZ*, *blaI*, *blaR*), genes involved in the resistance to Trimethoprim and Mupirocin (*dfrA* and *mupR*, respectively), a gene encoding unspecific efflux pump conferring resistance to a variety of antiseptic such as chlorhexidine (*qacA*), and genes potentially involved in virulence (*lukX*, *lukY*, *aur*). This variability affected at least 5 of the ten isolates.

## Conclusion

Our results indicate the gain/loss of resistance and virulence genes during a local outbreak, suggesting that the biological characteristics of the strain might vary through time.

## Disclosure of interest

None declared.

